# Oxidative stress in the aging substantia nigra and the etiology of Parkinson's disease

**DOI:** 10.1111/acel.13031

**Published:** 2019-08-20

**Authors:** Benjamin G. Trist, Dominic J. Hare, Kay L. Double

**Affiliations:** ^1^ Brain and Mind Centre and Discipline of Pharmacology, Faculty of Medical and Health The University of Sydney Sydney NSW Australia; ^2^ The Florey Institute of Neuroscience and Mental Health The University of Melbourne Parkville Vic. Australia; ^3^ Elemental Bio‐imaging Facility University of Technology Sydney Broadway NSW Australia

**Keywords:** antioxidant, oxidative stress, Parkinson's disease, reactive oxygen species

## Abstract

Parkinson's disease prevalence is rapidly increasing in an aging global population. With this increase comes exponentially rising social and economic costs, emphasizing the immediate need for effective disease‐modifying treatments. Motor dysfunction results from the loss of dopaminergic neurons in the substantia nigra pars compacta and depletion of dopamine in the nigrostriatal pathway. While a specific biochemical mechanism remains elusive, oxidative stress plays an undeniable role in a complex and progressive neurodegenerative cascade. This review will explore the molecular factors that contribute to the high steady‐state of oxidative stress in the healthy substantia nigra during aging, and how this chemical environment renders neurons susceptible to oxidative damage in Parkinson's disease. Contributing factors to oxidative stress during aging and as a pathogenic mechanism for Parkinson's disease will be discussed within the context of how and why therapeutic approaches targeting cellular redox activity in this disorder have, to date, yielded little therapeutic benefit. We present a contemporary perspective on the central biochemical contribution of redox imbalance to Parkinson's disease etiology and argue that improving our ability to accurately measure oxidative stress, dopaminergic neurotransmission and cell death pathways in vivo is crucial for both the development of new therapies and the identification of novel disease biomarkers.

## 1 INTRODUCTION

**Figure 1:**
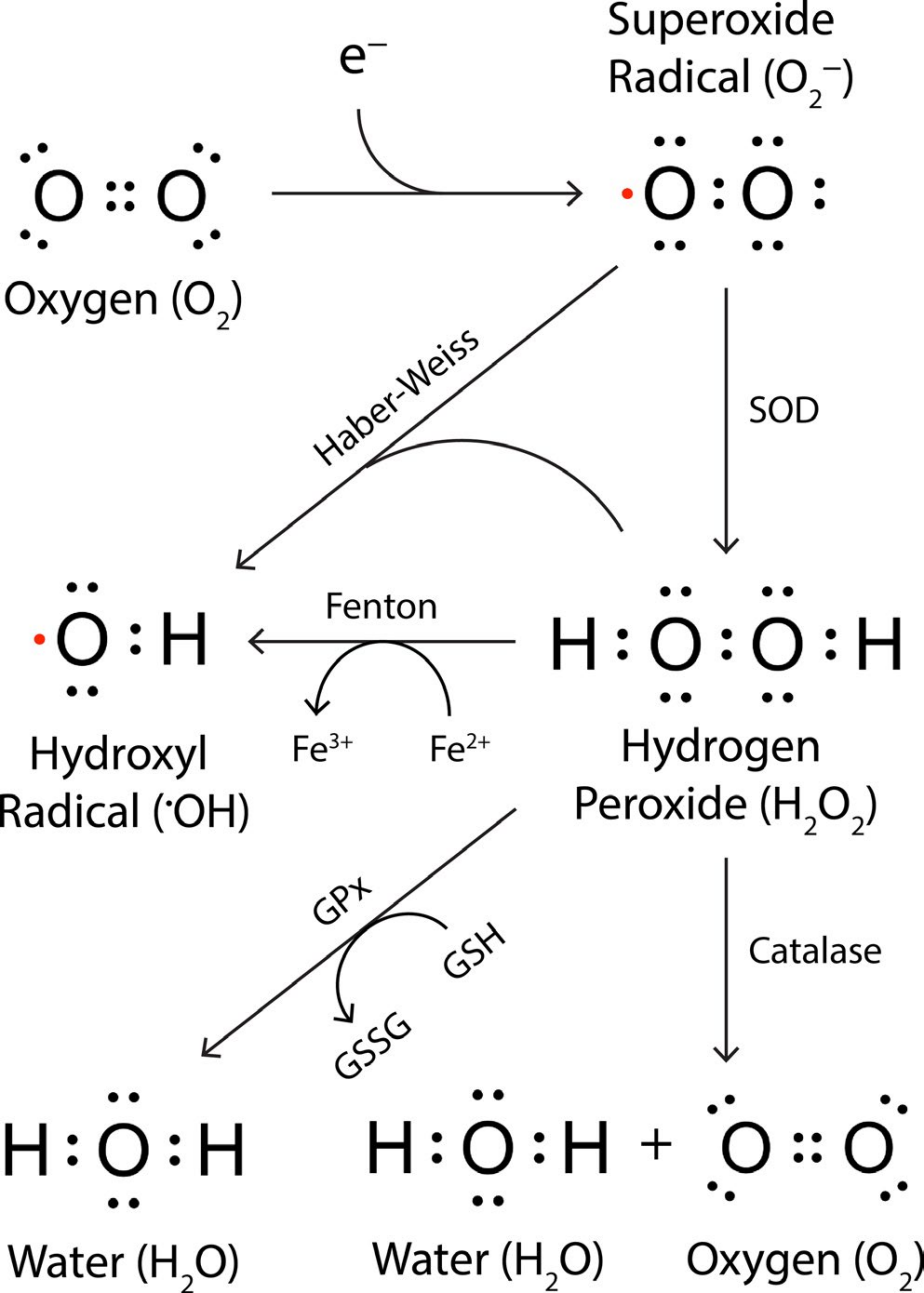
Common reactive oxygen species, their production, and clearance. Incomplete reduction of molecular oxygen (O_2_) produces superoxide radicals (O_2_^−^), which may be converted to hydroxyl radicals (^●^OH) via Haber–Weiss chemistry, or to hydrogen peroxide (H_2_O_2_) through the action of enzymes or molecules with superoxide dismutase (SOD) activity. Hydrogen peroxide is also a substrate for hydroxyl radical production via Fenton chemistry, catalyzed by labile ferrous iron. Hydrogen peroxide decomposition to water and oxygen is mediated by the enzymatic action of glutathione peroxidase (GPx) coupled to redox cycling of reduced (GSH) and oxidized (GSSG) glutathione, and also by catalase. Unpaired electrons are highlighted in red

Parkinson's disease (PD) is the most common neurodegenerative movement disorder. The global prevalence of PD is predicted to double by 2,040 (Dorsey & Bloem, 2018), making it the fastest growing neurodegenerative disorder ahead of Alzheimer's disease (Feigin et al., 2017). For perspective, if PD were transmissible, it would now be considered a global pandemic (Dorsey & Bloem, 2018). Movement dysfunction in PD results from the progressive death of dopaminergic neurons in the substantia nigra pars compacta (SNc), and accumulating evidence implicates oxidative stress as a key driver of the complex degenerating cascade underlying dopaminergic neurodegeneration in all forms of PD (Blesa, Trigo‐Damas, Quiroga‐Varela, & Jackson‐Lewis, 2015; Dias, Junn, & Mouradian, 2013). Oxidative stress arises from dysregulation of cellular redox activity, where production of reactive oxygen species (ROS; Figure 1) outweighs clearance by endogenous antioxidant enzymes and molecular chaperones. Oxidative stress in itself is therefore not pathological; rather, ROS accumulation following cellular redox imbalance mediates neuronal damage. Although ROS constitute important signaling molecules regulating physiological gene transcription and protein interactions (Schieber & Chandel, 2014), ROS accumulation can result in oxidative damage to lipids, proteins, DNA, and RNA depending on the subcellular location of ROS production, compromising neuronal function and structural integrity (Schieber & Chandel, 2014). Importantly, data collected from early‐stage PD patients demonstrate that elevated oxidative stress is a robust feature of initial disease stages, occurring prior to significant neuron loss (Ferrer, Martinez, Blanco, Dalfo, & Carmona, 2011). This implicates uncontrolled ROS generation as a potential causative factor in dopaminergic neuron death, rather than being a secondary response to progressive neurodegeneration. A better understanding of the complex role oxidative stress plays in the etiology of PD may therefore reveal new targets for therapeutic modification and preclinical diagnosis.

## 2 REDOX ENVIRONMENT WITHIN THE SUBSTANTIA NIGRA PARS COMPACTA

Specific subpopulations of dopaminergic neurons exist within the human SNc. In primates, dopamine neurons in the dorsal tier of the SNc (dSNc) receive projections from, and project to, the caudate and anterior putamen, which are themselves innervated by association cortices (Haber, Fudge, & McFarland, 2000). Ventral tier (vSNc) dopamine neurons, on the other hand, receive innervation from the caudate and anterior, intermediate, and posterior putamen and almost exclusively reciprocally innervate the intermediate and posterior putamen. In PD, significant degeneration of dopamine neurons is observed within both nigral subpopulations; however, neuronal loss in the vSNc precedes that observed within the dorsal tier, and is comparatively much more severe (Gibb & Lees, 1991). Identifying etiological factors for PD which differentiate the ventral and dorsal tiers of the SNc will greatly advance our understanding of the specific spatiotemporal progression of dopamine neuron death in this disorder.

Like many other neurodegenerative disorders, the biggest risk factor for PD is age: Sporadic PD is rare prior to 50 years; however, prevalence steadily increases to 2% in the global population aged 65 years, peaking at 5% in individuals aged 80 years (Tysnes & Storstein, 2017). This association suggests that age‐related biomolecular changes within brain regions that are vulnerable to degeneration in PD, namely the SNc, contribute to an increased risk of developing PD. Indeed, current data demonstrate moderate pathological change in the healthy postmortem human SNc compared with other similarly‐aged brain regions, including mild mitochondrial dysfunction, calcium, and iron dysregulation, and antioxidant deficiencies (James et al., 2015; Reeve, Simcox, & Turnbull, 2014; Venkateshappa et al., 2012). These pathologies are likely a product of disturbances in the unique biochemical environment within aging nigral dopaminergic neurons, which will be discussed in detail below, and are suggested to underlie the gradual shift in neuronal redox balance to dangerous levels as the brain ages. Importantly, age‐related redox changes within the SNc appear to manifest within the ventral tier more severely, indicating heightened redox dyshomeostasis within this nigral subregion may underlie its selective vulnerability. Improving our knowledge of oxidative pathology in the aging SNc may therefore enhance our understanding of both the origins of oxidative stress in PD, and the contribution of such processes to the spatiotemporal progression of SNc dopaminergic neurodegeneration in this disorder.

While oxidative stress is typically associated with neuron death, it is unclear whether mild and progressive ROS accumulation in the aging SNc results in gradual nigral dopaminergic neuron death in healthy individuals. Mild–moderate reductions (5%–10% per decade) in dopaminergic neuron density are reported in the postmortem SNc of approximately one‐third of clinically healthy, aged individuals (Buchman et al., 2012; Fearnley & Lees, 1991; Ma, Roytt, Collan, & Rinne, 1999), and dopamine receptor levels steadily decline (10% per decade) from early adulthood (Mukherjee et al., 2002). These results, however, are difficult to interpret given they do not account for the proportionate (5%) reduction in human brain volume per decade during aging (Svennerholm, Bostrom, & Jungbjer, 1997). Irrespective of its impact on neuronal survival during healthy aging, the high basal level of oxidative stress within aging SNc dopamine neurons is thought to confer vulnerability to oxidative insult following further deterioration of neuronal oxidative balance in PD. Many drivers of nigral oxidative stress in healthy aging have been identified as key contributors to heightened oxidative stress in the PD SNc, suggesting that the etiology of PD may involve an exacerbation of molecular pathways involved in healthy aging (Collier, Kanaan, & Kordower, 2017; Reeve et al., 2014). It is the presence of additional and compounding factors specifically in the PD SNc, however, that are thought to progressively exacerbate the imbalance between ROS production and clearance in this brain region in PD, associated with severe nigral neurodegeneration which is absent in healthy aging. Identifying prominent sources of ROS within the PD SNc may enable the development of targeted therapeutic approaches for PD, which mitigate SNc dopamine neuron loss by restoring redox homeostasis in this brain region.

## 3 MITOCHONDRIA

**Figure 2:**
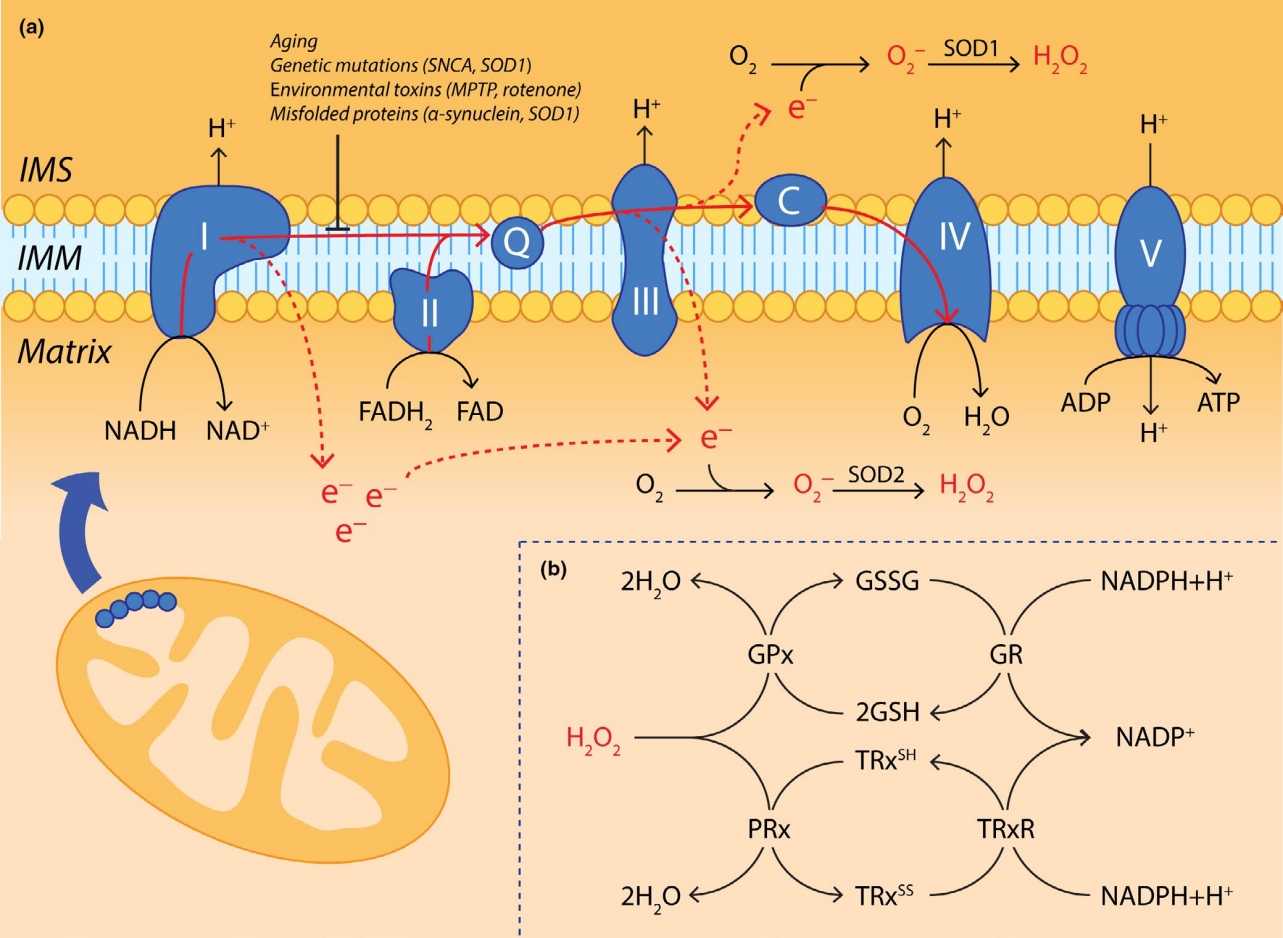
Reactive oxygen species are an inherent by‐product of oxidative phosphorylation in the mitochondrial ETC. (a) Electrons generated by the tricarboxylic acid cycle in the mitochondrial matrix are shuttled to ETC complexes I and II by NADPH and FADH_2_, respectively. They are then transferred to complex IV of the ETC with the help of inner mitochondrial membrane (IMM) electron shuttles (Q, coenzyme Q; C, cytochrome c) where they reduce molecular oxygen to water, a process which simultaneously drives ATP production by ATP synthase (ETC complex V). A small amount of premature electron leakage occurs naturally during oxidative phosphorylation, whereby electrons bound within ETC complexes I and III diffuse into both the mitochondrial matrix and intermembrane space (IMS). Here, they may cause incomplete reduction of molecular oxygen (O_2_), generating superoxide radicals (O_2_^−^) that may subsequently be converted to hydrogen peroxide (H_2_O_2_) through the action of superoxide dismutase 1 or 2 (SOD1/2). Electron leakage from the electron transport chain is worsened during healthy aging, or by pathogenic factors such as genetic mutations (*SNCA*, *SOD1*), environmental toxins (MPTP, rotenone), or misfolded proteins (α‐synuclein, SOD1). (b) Mitochondrial H_2_O_2_ levels are regulated by glutathione peroxidase (GPx) and peroxiredoxin (PRx), coupled to the redox cycling of glutathione (GSH/GSSG) and thioredoxin (TRx^SH^/TRx^SS^), respectively. While the oxidation component of each cycle is mediated by GPx and PRx, glutathione reductase (GR) and thioredoxin reductase (TRxR) drive NADPH‐dependent glutathione and thioredoxin reduction, respectively, to complete the redox loop

Mitochondria are a primary intracellular source of ROS during healthy aging (Brand et al., 2004). Mitochondrial ATP production powers neural activity and maintains cellular homeostasis, which is achieved through oxidative phosphorylation in the mitochondrial electron transport chain (ETC; Figure 2). Premature electron transfer from complexes I and III of the ETC to O_2_ occurs naturally in intact mammalian mitochondria (Drose & Brandt, 2008; Kussmaul & Hirst, 2006) and generates superoxide radicals (O_2_^−^) as a physiological by‐product of energy production. These ROS can trigger the formation of hydroxyl radicals (^●^OH), which are thought to mediate primary neuronal oxidative damage both within and outside of mitochondria following their diffusion out of mitochondria (Weidinger & Kozlov, 2015). As a counterbalance, mitochondria contain two of the three eukaryotic superoxide dismutases (SOD), which detoxify O_2_^−^ into less harmful hydrogen peroxide (H_2_O_2_; Ruszkiewicz & Albrecht, 2015). Manganese superoxide dismutase (SOD2) is localized to the mitochondrial matrix and inner membrane (Karnati, Luers, Pfreimer, & Baumgart‐Vogt, 2013), whereas copper/zinc superoxide dismutase (SOD1) exists within the mitochondrial intermembrane space, cytosol, and many other cellular compartments (Kawamata & Manfredi, 2010). Mitochondrial H_2_O_2_ produced by SOD1/2 is decomposed to innocuous O_2_ and H_2_O via specific mitochondrial glutathione peroxidases (GPx1/4; Brigelius‐Flohe & Maiorino, 2013) and peroxiredoxins (PRx3/5; Ruszkiewicz & Albrecht, 2015; Figure 2). While H_2_O_2_ likely represents less of a threat for oxidative damage compared with more redox‐active ROS, such as ^●^OH, the longer half‐life of H_2_O_2_ and greater rate of diffusion from mitochondria into other cellular compartments allow it to act as an effective redox signaling molecule (Collins et al., 2012; Murphy, 2009). This involves reversible oxidative modification of proteins, especially thiol groups of cysteine residues (Eaton, 2006), which act as a redox switch by altering physiological protein functions, promoting alternative protein functions, or facilitating secondary interactions (D'Autreaux & Toledano, 2007; Eaton, 2006; Murphy, 2009). Effective regulation of mitochondrial H_2_O_2_ by endogenous antioxidant pathways therefore constitutes an essential mechanism for maintaining physiological redox signaling and homeostasis.

### 3.1 Energy production and pacemaker activity in the substantia nigra pars compacta

The inherent production of ROS during ATP synthesis is greater within specific neuronal populations with higher energy demands, including dopaminergic neurons of the SNc. The large and unmyelinated axonal arbor of these neurons, whose size and complexity are orders of magnitude greater than other classes of dopamine neurons and other types of neurons in the brain (Pissadaki & Bolam, 2013), necessitates higher rates of ATP production to maintain resting membrane potential, propagate action potentials, and enable synaptic transmission. Unlike the majority of neurons throughout the brain, adult SNc dopamine neurons are also autonomously active. Regular action potentials (2–4 Hz) are generated in the absence of synaptic input (Grace & Bunney, 1983) to maintain dopamine levels in regions innervated by the SNc, especially the striatum (Romo & Schultz, 1990). Similar pacemaker function in neighboring neuronal populations, such as dopamine neurons of the ventral tegmental area, is driven by monovalent cation channels; however, SNc dopamine neurons employ L‐type Ca_v_1.3 Ca^2+^ channels (Bonci, Grillner, Mercuri, & Bernardi, 1998; Chan et al., 2007). Resulting Ca^2+^ influxes have the capacity to disrupt cell signaling and metabolic pathways, due to the importance of Ca^2+^ as a secondary messenger. Accordingly, imported Ca^2+^ must be immediately sequestered into the endoplasmic reticulum or mitochondria for storage, or pumped back into the extracellular space against an enormous concentration gradient, processes which require substantial amounts of energy (Perier & Vila, 2012). This constant Ca^2+^ buffering burden is a unique feature of SNc dopamine neurons, as other neuronal populations expressing Ca_v_1.3 Ca^2+^ channels are only activated episodically (Olson et al., 2005), making the task and metabolic cost of Ca^2+^ homeostasis relatively manageable. Together, the natural physiology and Ca^2+^ pacemaking activity of SNc dopamine neurons impose a high metabolic burden specifically on this neuronal population, which creates susceptibility to excessive ROS generation in situations when energy demand (protonmotive force) exceeds the capacity for supply. Mitochondrial ETC dysfunction, particularly in complex I (Puspita, Chung, & Shim, 2017), constitutes one such situation, whereby electron delivery to the ETC (demand) surpasses the redox capability of defective ETC complexes attempting oxidative phosphorylation (supply). The resultant increase in premature electron transfer to O_2_ generates substantial O_2_^−^, causing oxidative damage to DNA, lipids, and proteins both within and outside of mitochondria.

Mitochondrial complex I activity gradually diminishes within SNc dopamine neurons with age (Venkateshappa et al., 2012), and thus, the unique biophysical properties of nigral dopamine neurons may contribute to the high baseline level of oxidative stress within the aging SNc. Additionally, dopamine neurons within the healthy human vSNc do not express calbindin, an important neuronal Ca^2+^ buffering protein (Schmidt, 2012), contrast to substantial expression in the dSNc (Reyes et al., 2012). These data suggest Ca^2+^ dysregulation and redox imbalance related to aging or PD etiology may impact the ventral tier of the SNc well before the dorsal tier due to insufficient Ca^2+^ buffering, which may contribute to the selective vulnerability of the vSNc to degeneration in PD. Indeed, nigral dopamine neurons lacking calbindin are selectively vulnerable to degeneration following administration of the mitochondrial toxin 1‐methyl‐4‐phenyl‐1,2,3,6‐tetrahydropyridine (MPTP) in nonhuman primates (German, Manaye, Sonsalla, & Brooks, 1992), and this can be rescued by viral vector‐mediated recruitment of calbindin into these neurons (Inoue et al., 2018).

Overall, maintaining Ca^2+^ pacemaker activity throughout such a complex axonal architecture imposes a tighter energy budget on nigral dopamine neurons compared with those in other brain regions, especially those within the vSNc, which is likely to contribute to their high baseline level of oxidative stress and vulnerability to degeneration in PD. Ca^2+^ dyshomeostasis, in particular, is likely to constitute a novel therapeutic target for PD, considering the substantial therapeutic benefit of bolstering Ca^2+^ buffering in the vSNc of nonhuman primates using calbindin. Multiple retrospective analyses have identified a significant reduction in risk of PD in hypertensive patients treated with Ca^2+^ channel antagonists, but not with other anti‐hypertensive medications (Becker, Jick, & Meier, 2008; Rodnitzky, 1999). Further, similar to calbindin, Ca^2+^ channel antagonists diminish the sensitivity of SNc dopamine neurons to mitochondrial toxins (Chan et al., 2007). Elicited reductions in Ca^2+^ influx do not appear to ameliorate physiological pacemaking activity, nor do they elicit learning or cognitive deficits in mice (Bonci et al., 1998). Accordingly, a phase III, double‐blind, placebo‐controlled, randomized clinical trial of an L‐type Ca^2+^ channel blocker is currently underway in a large cohort of early PD patients (NCT02168842).

### 3.2 Mitochondrial dysfunction

While increasing mitochondrial ROS production in the aging SNc arises from the gradual deterioration of physiological redox regulation, excessive ROS generation by mitochondria in the PD SNc is associated with severe ETC impairment and oxidative damage imposed by additional environmental toxins and pesticides, and genetic mutations (Schapira et al., 1990). These compounding elements are likely to drive an already energetically stressed mitochondrial ETC system past its absolute redox capability, combining with the high energy demands of a complex axonal arbor and large calcium‐buffering burden to trigger a severe disequilibrium in electron delivery and utilization by the ETC. A number of environmental toxins and pesticides, including MPTP and rotenone, freely cross lipid membranes and accumulate in mitochondria following inhalation or ingestion (Perier, Bove, Vila, & Przedborski, 2003). Once inside mitochondria, they significantly impair mitochondrial complex 1 redox activity by blocking the flow of electrons from NADH dehydrogenase to coenzyme Q (Ramsay et al., 1991; Richardson, Quan, Sherer, Greenamyre, & Miller, 2005), promoting significant O_2_^−^ generation and reducing ATP synthesis. Importantly, MPTP treatment only yields a transient 20% reduction in mouse striatal and midbrain ATP levels in vivo (Chan, DeLanney, Irwin, Langston, & Di Monte, 1991), suggesting MPTP‐induced ATP deficiency may not play a primary pathogenic role in PD. Altered mitochondrial function per se cannot be automatically equated with energy failure, reduced energy function impairing optimal neuron functioning but still compatible with survival (Pathak, Berthet, & Nakamura, 2013). Nonetheless, these compounds demonstrate acute and preferential cytotoxicity to nigral dopaminergic neurons (Blesa & Przedborski, 2014). It is therefore more likely that other MPTP‐induced biochemical changes, most notably significant ROS generation, contribute to the selective vulnerability of this unique neuronal population to degeneration in PD. Supporting a role for ROS in MPTP‐induced neurotoxicity, transgenic mice with increased SOD1 antioxidant activity are resistant to dopaminergic denervation following MPTP administration (Przedborski et al., 1992). Admittedly, these compounds are rare outside of a laboratory environment; however, understanding the bases of their preferential toxicity to nigral dopaminergic neurons will undoubtedly uncover important mechanisms underlying specific SNc neurodegeneration in PD.

In addition to environmental chemical factors, nearly all known genetic mutations linked to PD result in an impairment of mitochondrial complex I activity and associated ROS production, albeit via different molecular pathways. Generally, mutations associated with autosomal recessive forms of PD (*DJ‐1*, *PINK‐1*, *PARK2*, *GBA‐1*, *ATP13A2*) result in mitochondrial fragmentation and loss of complex I activity following a loss of function of their protein products (Blesa et al., 2015; Dias et al., 2013; Gegg & Schapira, 2016; Gusdon, Zhu, Van Houten, & Chu, 2012; Hayashi et al., 2009; Muftuoglu et al., 2004), indicative of an important role for these proteins in the physiological function of the mitochondrial ETC. Accordingly, introduction of human wild‐type DJ‐1 or PINK‐1 can rescue complex I deficiency in transgenic strains of *D. melanogaster* or mice expressing human mutant DJ‐1 or PINK‐1 proteins (Hao, Giasson, & Bonini, 2010; Morais et al., 2009). The endogenous function of DJ‐1 may also protect against ROS generated by the calcium pacemaking activity of SNc dopamine neurons under physiological conditions (Guzman et al., 2010). In contrast, mutations underlying autosomal dominant PD (*SNCA, LRRK2*) are associated with a gain‐of‐toxic function of misfolded protein products, disrupting mitochondrial oxidative phosphorylation via an interaction with complex I. The mitochondrial accumulation of mutant (Martin et al., 2006) or wild‐type (Hsu et al., 2000) α‐synuclein, the protein product of *SNCA,* in dopaminergic neurons reduces complex I activity and elevates ROS production in vivo in transgenic mice. This occurs prior to striatal dopamine loss (Subramaniam, Vergnes, Franich, Reue, & Chesselet, 2014), strongly suggestive of a causative role in dopaminergic neuron death in human PD. The specific vulnerability of SNc dopaminergic neurons in familial PD, despite the system‐wide presence of genetic mutations throughout the PD brain and body, again highlights the unique susceptibility of this neuronal population to damage following increased mitochondrial oxidative stress.

## 4 DOPAMINE AND IRON REDOX CHEMISTRY

**Figure 3:**
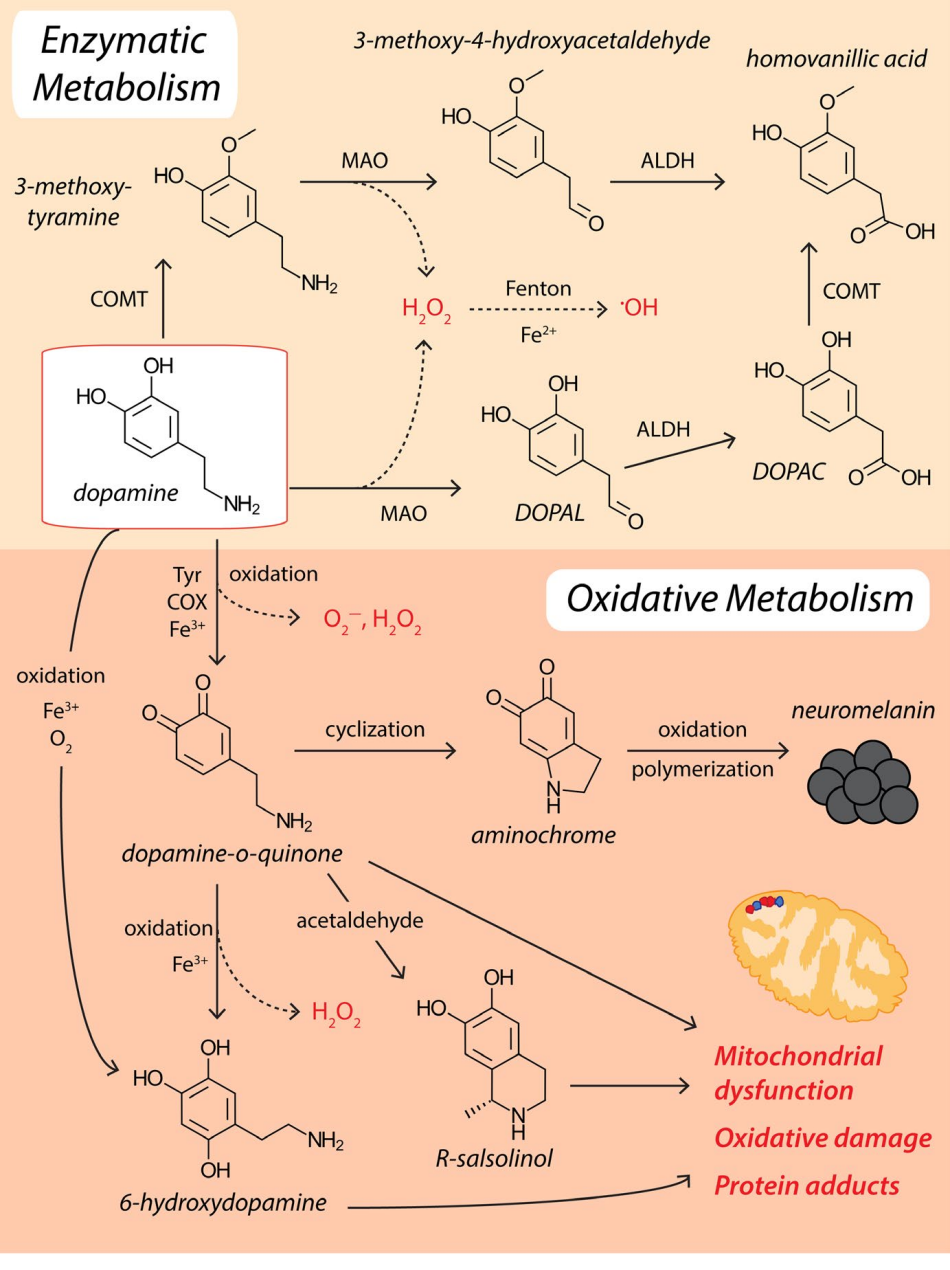
Dopamine metabolism and ROS production. Enzymatic decomposition of dopamine to homovanillic acid is mediated by monoamine oxidase (MAO), catechol‐o‐methyl transferase (COMT), and aldehyde dehydrogenase (ALDH). Conversely, dopamine may be oxidized to dopamine‐o‐quinone by tyrosinase (Tyr), cyclooxygenase (COX), or labile ferric iron (Fe^3+^). Dopamine‐o‐quinones are reactive intermediates for the generation of more damaging compounds, including 6‐hydroxydopamine and R‐Salsolinol. Endogenous detoxification of dopamine‐o‐quinones involves cyclization to produce aminochrome, and subsequent oxidation and polymerization to generate neuromelanin. Hydrogen peroxide (H_2_O_2_) produced by MAO, dopamine oxidation and dopamine‐o‐quinone oxidation, can participate in Fenton chemistry and react with labile ferrous iron (Fe^2+^) to generate damaging hydroxyl radicals (^●^OH)

Significant amounts of ROS are generated within SNc dopaminergic neurons and surrounding glia by the oxidative metabolism of dopamine (Westlund, Denney, Rose, & Abell, 1988; Figure 3). Oxidative deamination of dopamine by monoamine oxidases generates H_2_O_2_ as a by‐product, whereas enzymatic oxidation of dopamine's electron‐rich catechol moiety by cyclooxygenases, tyrosinase, and other enzymes produces O_2_^−^ (Muñoz, Huenchuguala, Paris, & Segura‐Aguilar, 2012). Auto‐oxidation of dopamine may also occur via interactions with labile iron and other biometals (Hare & Double, 2016), generating ROS (H_2_O_2_, O_2_^−^, ^●^OH), pro‐oxidant dopamine‐o‐quinones (DAQ) and a raft of other neurotoxins (Figure 3), comprehensively reviewed elsewhere (Hare & Double, 2016; Sun, Pham, Hare, & Waite, 2018). Importantly, specific pathways within this complex network of iron–dopamine chemistry are seemingly favored by different chemical environments in vitro. Acidosis accelerates dopamine oxidation and promotes aminochrome formation, suggesting clinically measured brain tissue acidosis in PD may promote iron–dopamine redox chemistry and the accumulation of pro‐oxidant aminochrome in the SNc in this disorder (Sun et al., 2018). While these investigations have yet to be translated into complex cellular systems, they may have the potential to underlie the progressive and worsening nature of cell loss in PD.

Of the iron–dopamine metabolites, DAQs constitute particularly versatile intermediates in pathways producing harmful pro‐oxidant dopamine derivatives, aside from their own capacity to alkylate protein thiol and amine groups and promote protein oxidation in the presence of ROS (Meiser, Weindl, & Hiller, 2013). The tetrahydroisoquinoline salsolinol is one such DAQ derivative, which enhances oxidative stress and mitochondrial damage by inhibiting ETC function (Su et al., 2013). Salsolinol also disrupts clearance of dopamine by monoamine oxidases (Napolitano, Manini, & d'Ischia, 2011), shifting dopamine metabolism toward more damaging metabolic pathways which produce DAQs. Another derivative, 6‐hydroxydopamine (6‐OHDA), generates substantial amounts of O_2_^−^ by inhibiting mitochondrial ETC complexes I and IV (Puspita et al., 2017). Given the enormous neurotoxic potential of DAQs, and the remarkably slow conversion of DAQs to neuromelanin (NM), it is likely endogenous mechanisms of DAQ detoxification exist. Potential roles for SOD1, glutathione transferase (by way of glutathione conjugation), and macrophage migration inhibitory factor have been suggested via evidence of direct interactions with DAQs (Emdadul Haque et al., 2003).

### 4.1 Iron accumulation

Pro‐oxidant interactions between iron and dopamine are suggested to be enhanced in the aging SNc because of a preferential accumulation of labile iron in this brain region (Hare & Double, 2016). This is perhaps associated with age‐dependent ferritin dysfunction documented in *Caenorhabditis elegans*, whereby reactive ferrous iron (Fe^2+^) is no longer efficiently oxidized to more chemically stable ferric iron (Fe^3+^) for storage (James et al., 2015). Similar experiments have not been performed in human postmortem tissues, owing to difficulties in preserving iron redox state during tissue collection procedures, as well as our inability to accurately assay ferritin iron loading in vivo. Further, it will be important to determine the relative contributions of microglia and astrocytes to iron accumulation in the aging SNc, as these non‐neuronal cell types typically store approximately three times the quantity of iron compared with neurons without exhibiting signs of iron‐mediated toxicity (Bishop, Dang, Dringen, & Robinson, 2011).

Iron accumulation in PD is significantly enhanced compared with healthy aging; iron levels are elevated twofold in the postmortem SNc compared with age‐matched controls (Dexter et al., 1989; Genoud et al., 2017). In vivo MRI imaging of SNc proton transverse relaxation states, known to be correlated with regional iron content, in early PD patients and age‐matched controls revealed iron deposition was limited to the vSNc in early‐stage PD (Martin, Wieler, & Gee, 2008). While these data indirectly suggest iron accumulation correlates with the specific spatiotemporal progression of neuron loss in PD, direct measurement of SNc iron content in the vSNc and dSNc of early PD patients in vivo or postmortem will be necessary to implicate iron‐mediated toxicity in PD etiology with any great certainty. Investigations using cutting‐edge spatial imaging technologies, such as laser ablation‐inductively coupled plasma–mass spectrometry or synchrotron radiation X‐ray fluorescence microscopy (Hare, Kysenius, et al., 2017), are warranted.

The origin of this increase is unknown, but recent evidence suggests excessive early‐life iron intake in iron‐supplemented cereals and infant formulae may contribute to brain iron accumulation, and thus perhaps risk of PD (Hare, Cardoso, et al., 2017). The impact of high dietary iron intake during adolescent and adult life on the risk of developing PD is less clear (Logroscino, Gao, Chen, Wing, & Ascherio, 2008). Dysregulation iron metabolism in PD may also involve unregulated phosphorylation or oxidation of α‐synuclein (Duce et al., 2017), a well‐documented feature within the PD SNc (Barrett & Timothy Greenamyre, 2015). Atypical posttranslational modification of α‐synuclein triggers redistribution to the cytoplasm, which impairs transferrin receptor‐mediated iron import (Baksi, Tripathi, & Singh, 2016). This induces alternative iron import mechanisms, such as DMT1, which are not subject to negative‐feedback regulation by intracellular iron levels (Salazar et al., 2008), and may therefore contribute to intracellular iron accumulation in the PD SNc. An increase in DMT1 is observed in the SNc of PD patients (Salazar et al., 2008). Aside from excessive or unregulated iron intake, it is possible that alterations in the iron storage protein ferritin contribute to heightened iron accumulation in PD, although it is unclear if and how ferritin protein levels are altered, or whether reported reductions in the storage capacity of ferritin with age are exacerbated or improved in PD. Neuronal iron export, on the other hand, is reduced in the PD SNc via a reduction in the ferroxidase activity of ceruloplasmin, an essential cuproprotein mediating neuronal iron export through ferroportin onto interstitial apo‐transferrin (Ayton et al., 2013). Impairment of ceruloplasmin ferroxidase activity is associated with severe intraneuronal copper deficiency in the PD SNc (Davies et al., 2014; Genoud et al., 2017), which likely favors copper delivery to cuproproteins with high copper‐binding affinities (SOD1, cytochrome c oxidase) at the expense of ceruloplasmin. Additional reductions in amyloid precursor protein (Ayton et al., 2015) and soluble tau protein (Lei et al., 2012) in the PD SNc are also believed to destabilize ferroportin at the cell surface to impair iron export (Wong et al., 2014).

Together, current data indicate a multilayered failure of iron metabolism specifically within the PD SNc (New, 2013), resulting in an increased pool of pro‐oxidant, labile cytoplasmic iron in this brain region in PD. Importantly, other brain regions also exhibit signs of iron accumulation in PD (Wang et al., 2016), and thus, iron accumulation alone is unable to explain the specific vulnerability of the SNc to degeneration in PD. Additional factors must be present in this brain region which potentiate the toxicity of iron selectively in the SNc (Genoud et al., 2017).

### 4.2 Dopamine transporters, α‐synuclein, and neurotransmitter release

**Figure 4:**
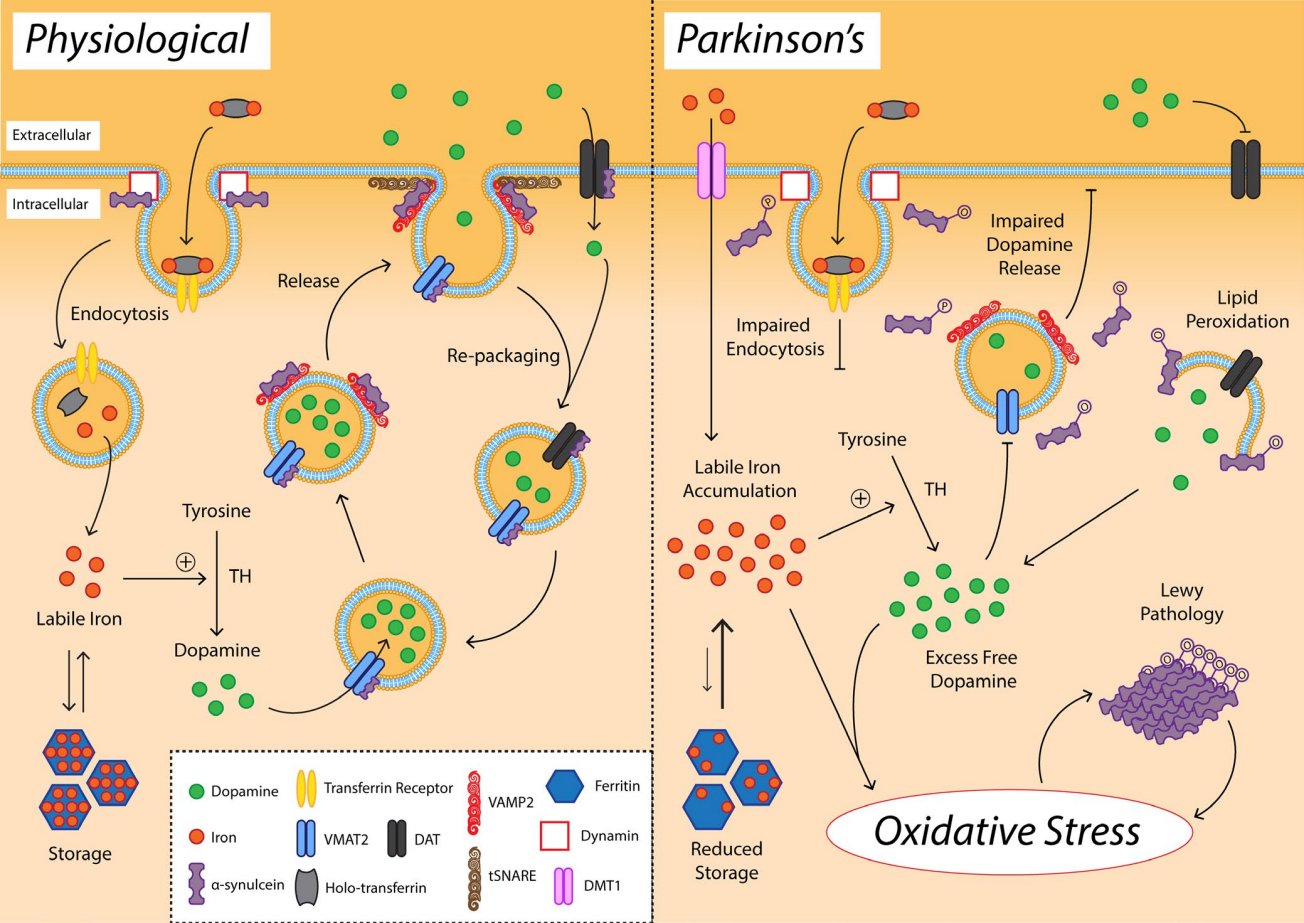
Alterations in dopamine, iron, and α‐synuclein promote oxidative stress selectively in the SNc. Under physiological conditions, α‐synuclein facilitates dynamin‐mediated endocytosis of transferrin receptor and iron‐bound holo‐transferrin. A facile cytoplasmic labile iron pool is tightly maintained by ferritin to enable ferroprotein function, including dopamine production by tyrosine hydroxylase (TH). α‐Synuclein facilitates multiple steps in synaptic dopamine release and repackaging, including VMAT2‐mediated dopamine packaging into synaptic vesicles, VAMP2 binding to tSNARE proteins in the presynaptic membrane, and dopamine transporter (DAT)‐mediated synaptic dopamine reuptake and repackaging into synaptic vesicles. In Parkinson's disease, oxidation and phosphorylation of α‐synuclein impair transferrin receptor‐mediated iron import, necessitating the utilization of divalent metal transporter 1 (DMT1), which is not regulated by intracellular iron levels. Combined with an age‐dependent diminution in the iron storage capacity of ferritin, this elevates the labile iron pool, which participates in Fenton chemistry and reacts with free dopamine to produce ROS. Free dopamine is also elevated due to impaired dopamine packaging into synaptic vesicles and reduced synaptic dopamine release, both of which are associated with atypical posttranslational modification of α‐synuclein. Oxidation and phosphorylation of α‐synuclein is associated with Lewy pathology deposition, exacerbating nigral oxidative stress. Figure adapted from Duce et al. (2017)

Dopamine transporter (DAT) and vesicular monoamine transporter 2 (VMAT2) represent major defense mechanisms against ROS generated by iron–dopamine chemistry, removing free dopamine from the synapse and packaging it into synaptic vesicles where it is comparatively protected from oxidation (Exner, Lutz, Haass, & Winklhofer, 2012; Figure 4). Nigral expression of DAT, but not VMAT2, appears to gradually decline with age (Ma, Ciliax, et al., 1999), suggesting that reduced clearance of synaptic dopamine may augment ROS production in the SNc during healthy aging by promoting oxidative metabolism of free dopamine. Wild‐type α‐synuclein is known to interact with VMAT2 during vesicle filling (Yavich, Tanila, Vepsalainen, & Jakala, 2004) and to inhibit DAT‐mediated synaptic dopamine reuptake (Butler et al., 2015) and is proposed to play a physiological role in both processes (Figure 4). The fusion and clustering of tSNARE‐associated vesicles to the presynaptic membrane is also regulated by an interaction of α‐synuclein with VAMP2 in the presynaptic terminal (Burre et al., 2010), which keeps VAMP2 in close proximity to tSNAREs to regulate neurotransmitter release.

**Figure 5:**
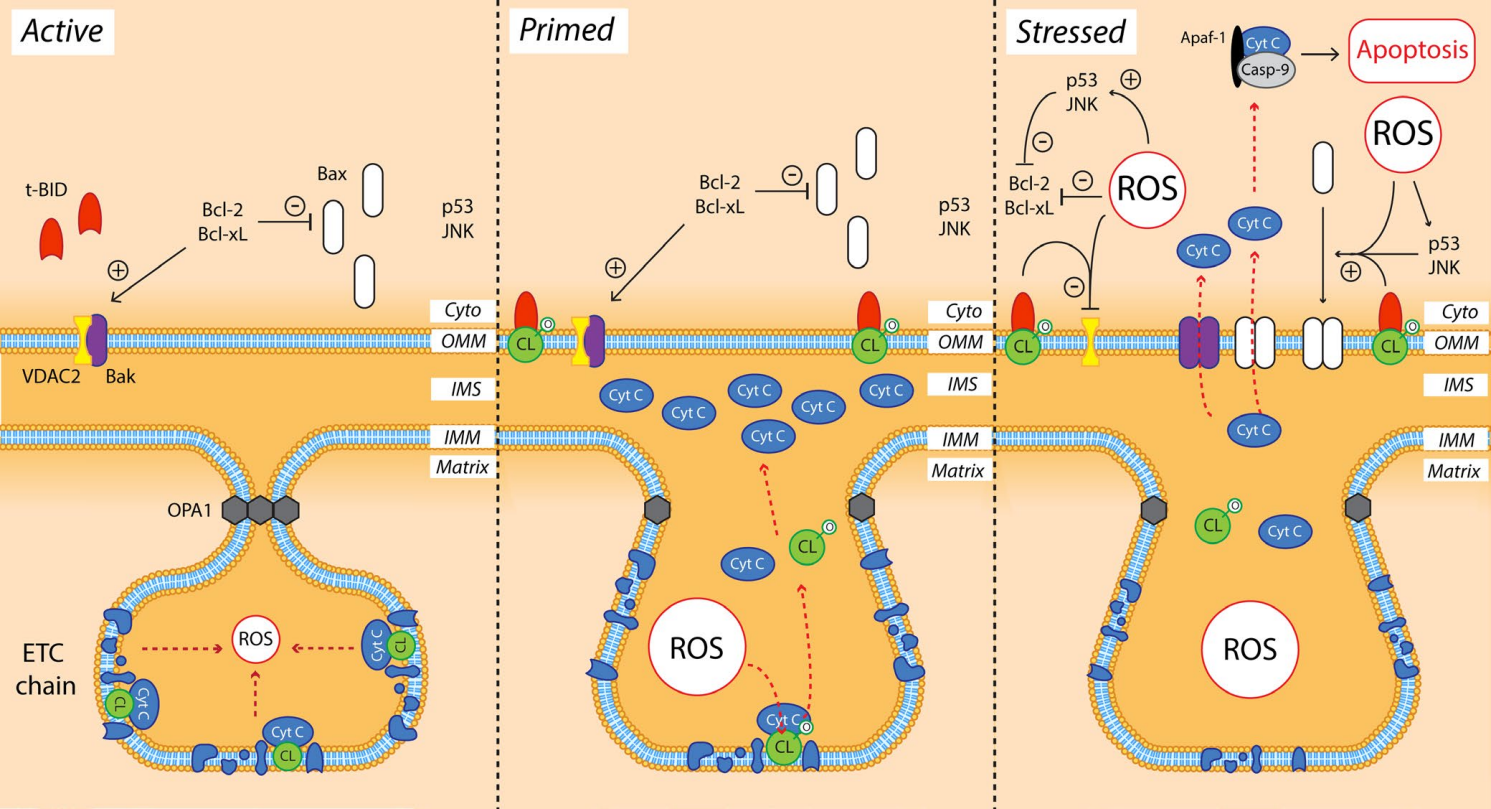
ROS‐dependent priming and activation of the intrinsic cell death pathway. In active mitochondria, cardiolipin (CL) anchors cytochrome c (Cyt C) in the inner mitochondrial membrane (IMM) between complexes III and IV of the ETC. The structure of inner mitochondrial membrane cristae junctions is maintained by OPA1 oligomers. Anti‐apoptotic Bcl‐2 family proteins (Bcl‐2, Bcl‐xL) dominate over pro‐apoptotic Bcl‐2 family proteins (Bak, Bax) preventing outer mitochondrial membrane (OMM) permeabilization and the induction of apoptosis. VDAC2 binding also inhibits Bak activation. Toxins such as MPTP and Rotenone (and possibly genetic mutations; *Parkin, PINK‐1*) induce significant mitochondrial oxidative stress, which triggers cristae remodeling via OPA1 oligomer dissociation, and catalyzes the disconnection of Cyt C from CL following oxidation of CL. Oxidized CL relocates to the OMM, where it binds cytoplasmic truncated (t)‐BID, and Cyt C accumulates in the intermembrane space (IMS). This does not trigger the intrinsic cell death pathway, but is thought to prime mitochondria for the release of Cyt C and other pro‐apoptotic factors (Smac, AIF; not shown) upon compounding cellular stress signals. Additional oxidative stress impairs Bcl‐2/Bcl‐xL and VDAC2 and activates p53/JNK and Bax/Bak, causing translocation of Bax/Bak to the OMM with the help of outer membrane CL‐BID complexes. This triggers OMM permeabilization and the release of Cyt C, Smac, and AIF. In the cytoplasm (Cyto), Cyt C binds to Apaf‐1 and procaspase‐9, which together activate executioner caspases that trigger apoptosis

In the PD SNc, surviving dopaminergic neurons exhibit an increase in dopamine production, and a concomitant reduction in synaptic dopamine clearance and repackaging into vesicles, owing to dysregulation of DAT and VMAT2 within these neurons (Harrington, Augood, Kingsbury, Foster, & Emson, 1996; Nutt, Carter, & Sexton, 2004). Alterations to VMAT2 and DAT are associated with aberrant posttranslational modification or mutation of α‐synuclein protein, which can impede VMAT2‐mediated repackaging of dopamine into synaptic vesicles (Lotharius & Brundin, 2002), and impair regulation of DAT expression at the cell surface (Sidhu, Wersinger, & Vernier, 2004). Combined, these changes elevate free cytoplasmic dopamine, promoting iron–dopamine redox chemistry and the production of DAQs and 6‐OHDA, which are more neurotoxic redox species than ^●^OH (Zhou, Lan, Tan, & Lim, 2010; Figures 4 and 5).

The dysregulation of iron and dopamine metabolism within the PD SNc is intrinsically linked to pathological α‐synuclein protein, and the convergence of these factors specifically within the SNc may contribute to its selective vulnerability in PD. The formation of toxic products from iron–dopamine chemistry in the SNc produces a far more damaging redox environment in this brain region compared with nondopaminergic brain regions exhibiting iron accumulation or α‐synuclein pathology in isolation. Counteracting the formation of these damaging redox species constitutes a primary target for therapeutic interventions aiming to mitigate oxidative stress within the PD SNc, and conservative iron chelation is already being trialed in early‐stage PD patients (NCT02655315) following promising results in animal models of PD (Devos et al., 2014).

## 5 NEUROMELANIN

Neuromelanin is a complex biopigment composed of 35% lipids (primarily dolichol; Fedorow, Pickford, et al., 2006), 15% covalently bound peptides, and numerous products of catecholamine metabolism, explaining its spatial distribution within select populations of catecholamine neurons within the brain (Double et al., 2008). Neuromelanin accumulates in the cytoplasm as large amorphous granules of an inconsistent size (Fedorow, Pickford, et al., 2006), contrast to the regular spherical macrostructure of peripheral melanins. Optical density and area measurements of unstained NM in the postmortem SNc identify three developmental phases, beginning with the initiation of pigmentation at approximately 3 years of age (Fedorow, Halliday, et al., 2006). Increases in pigment granule volume and pigment density within granules are subsequently observed until age 20; however, after this point, increases in pigment density within granules occur without substantial growth in pigment volume, suggesting regulation of NM production and turnover.

The exact mechanism of NM production in the human SNc is unclear; however, NM is considered a complex biopolymer associated with dopamine autoxidation, rather than enzymatic catalysis. Tyrosinase catalyzes the rate‐limiting step of peripheral melanin synthesis; however, despite tyrosinase mRNA expression in the human SNc (Xu et al., 1997), no tyrosinase protein has yet been identified in this region (Tribl, Arzberger, Riederer, & Gerlach, 2007). Conversely, in vitro oxidation of dopamine produces dopamine melanin (DAM), which exhibits a moderate degree of chemical similarity to human NM (Double et al., 2000). Artificial synthesis of DAM in mouse SNc cell cultures is heavily reliant on nonvesicular dopamine, as well as ferric iron (Sulzer et al., 2000). This may be explained by the comparative susceptibility of nonvesicular dopamine to oxidation, which is catalyzed by ferric iron, producing DAQs and aminochrome. The identification of these particular products of dopamine oxidation in human NM suggests a degree of translation of these findings to human NM formation (Smythies, 1996; Figure 3). Despite similarities between NM and DAM, however, human NM possesses a substantially more complex structure and biochemical composition (Double et al., 2000), suggesting oxidized neurotransmitter is not the sole component of human NM. Further, not all cells that produce dopamine contain NM (Gaspar et al., 1983), suggesting that NM production may either be induced or inhibited, or that a mechanism of NM clearance exists. Regulation of NM production and/or degradation is consistent with the constant volume of NM within mature SNc dopamine neurons past the age of 20 (Fedorow, Halliday, et al., 2006), as uncontrolled autoxidation of dopamine over subsequent decades would be expected to result in a linear increase in NM volume.

Functionally, NM is believed to bind, store, protect, and release free dopamine, regulate redox‐active iron to minimize pro‐oxidant Fenton chemistry, and sequester a range of potentially toxic metal cations (zinc, copper, manganese, chromium, cobalt, mercury, lead, and cadmium) and chemicals (derivatives of paraquat, salsolinol, MPTP; Haining & Achat‐Mendes, 2017). Neuromelanin therefore bears closer resemblance to a protective cellular scaffold, rather than merely an aggregate of metabolic products, which is capable of sequestering toxic chemicals away from cellular compartments where they can participate in damaging biochemical reactions. In this way, NM may play a key role in redox homeostasis in the healthy SNc, especially with regard to the mitigation of iron–dopamine redox chemistry. Indeed, human‐derived NM prevents iron‐mediated ROS generation and antioxidant depletion in vitro (Zecca, Casella, et al., 2008).

### 5.1 Neuromelanin in PD: a loss or gain of function?

Contrast to its protective role in the healthy SNc, alterations to NM density and composition are thought to exacerbate ROS generation, iron accumulation, and α‐synuclein aggregation in the PD SNc (Faucheux et al., 2003; Halliday et al., 2005). An early increase in NM density within pigment granules is reported within morphologically normal SNc dopamine neurons, which is associated with increased NM oxidation and iron loading (Faucheux et al., 2003). Both of these factors promote the concentration of α‐synuclein to the lipid component of NM at the expense of cholesterol (Halliday et al., 2005), and iron loading is also shown to potentiate peroxidation of human‐derived NM in vitro (Zecca, Casella, et al., 2008). The accumulation of α‐synuclein pathology on NM is associated with a significant reduction in NM density within SNc dopamine neurons (Faucheux et al., 2003; Halliday et al., 2005), suggesting early α‐synuclein redistribution to NM promotes its decomposition, which is likely to impair the neuroprotective function of NM in PD. Indeed, reductions in NM density are associated with elevated NM redox activity and ROS production in PD postmortem SNc, which are positively correlated with increased levels of redox‐active iron in tissue surrounding melanized neurons (Faucheux et al., 2003). These data indicate the early redistribution of α‐synuclein to NM promotes free iron accumulation and ROS generation in nigral dopamine neurons in PD, and are consistent with reductions in the iron content of NM in the postmortem SNc of end‐stage PD patients (Bolzoni et al., 2002).

Upon progressive SNc dopamine neuron degeneration in PD, iron‐loaded NM is released into the interstitium, where it likely becomes phagocytosed by microglia and decomposed in an H_2_O_2_‐dependent manner (Zecca, Casella, et al., 2008). This releases redox‐active iron and other toxic chemicals and proteins previously sequestered by NM, which has been shown to trigger microglial activation and ROS production (Zecca, Wilms, et al., 2008).

A greater understanding of the synthesis and regulation of NM in the SNc during healthy aging and PD will clearly advance our understanding of the unique biochemistry of nigral dopamine neurons, and may provide insight into the pathogenesis of PD. Further, a comparative deficiency of NM within the vSNc, compared with the dSNc, in the healthy brain (Gibb & Lees, 1991) suggests the vSNc is more susceptible to oxidative insult, and indicates NM distribution within the SNc may contribute to the preferential degeneration of vSNc dopamine neurons in PD.

## 6 ANTIOXIDANT DYSFUNCTION

Augmenting progressive ROS production within the aging SNc is an age‐dependent reduction in the levels and function of key antioxidants. A region‐specific decrease in the levels of reduced glutathione, and a reduction in SOD, GPx, and glutathione reductase activities are all reported in the postmortem SNc of healthy aged individuals compared with younger individuals (Venkateshappa et al., 2012). Additionally, age‐dependent reductions in mRNA expression and enzymatic activity of GRx, PRx, and TRx pathways have been documented in mouse and human non‐neuronal cell types (Lim & Luderer, 2011; Xing & Lou, 2010), although the presence of such changes within dopamine neurons of the human SNc has not been investigated. These deficiencies suggest a gradual diminution in the capacity of nigral dopamine neurons to offset rising ROS production as we age may contribute to the vulnerability of this brain region to oxidative insult in PD.

While the healthy SNc experiences moderate age‐dependent antioxidant decline, the PD SNc is characterized by severe and widespread antioxidant system deficits, which are thought to compound disease‐associated ROS production. A drastic reduction (~50%) in total glutathione and GPx activity in the SNc of PD patients reflects significant dysfunction of the glutathione/GPx system (Sian et al., 1994), which may be associated with severe copper deficiency in this brain region in PD (Davies et al., 2014). Glutathione production in the PD SNc is likely hindered by a substantial reduction in γ‐glutamylcysteine synthetase activity, responsible for the de novo synthesis of glutathione (Kang et al., 1999), which has been linked to mutations in the *DJ‐1* gene, or abnormal posttranslational modifications of DJ‐1 protein, in familial and sporadic PD (Zhou & Freed, 2005). A decrease in the levels of reduced glutathione in this brain region in PD (Sian et al., 1994) further indicates glutathione recycling is either impaired, or is unable to match cellular H_2_O_2_ production, diminishing its contribution to ROS detoxification in this disorder. H_2_O_2_ buildup in the PD SNc is compounded by a significant reduction in the levels and function of catalase in this brain region, compared with that in the healthy aged brain (Ambani, Van Woert, & Murphy, 1975). In addition to H_2_O_2_, O_2_^−^ clearance in the PD SNc may be diminished due to SOD1 enzymatic dysfunction and aggregation (Trist et al., 2017; Trist, Fifita, et al., 2018; Trist, Hare, & Double, 2018), which is also associated with neuronal copper deficiency and misfolded α‐synuclein in this brain region (Helferich et al., 2015).

Importantly, deficits in the glutathione/GPx system (Zeevalk, Razmpour, & Bernard, 2008) and in SOD1 protein (Trist et al., 2017) are also a feature of incidental Lewy body disease (ILBD), a pathologically defined disease state thought to represent preclinical PD (DelleDonne et al., 2008), indicating that these events occur during early‐stage PD prior to neuronal loss, and may play a causative role in PD etiology. Despite our limited understanding of mechanisms underlying widespread antioxidant decline in PD, it is clear that levels of essential biometals, such as copper, and genetic mutations both play key roles. A greater understanding of molecular pathways leading to antioxidant dysfunction in PD may enable us to develop therapies that restore the antioxidant buffering capacity of vulnerable dopaminergic neurons and attenuate neurodegeneration in PD.

### 6.1 Investigating the antioxidant contribution of glia

Astrocytes and microglia express a wider range of antioxidant genes at significantly higher levels compared with neurons (Baxter & Hardingham, 2016), including SOD1/2, catalase, GPx, PRx, and TRx (Vilhardt, Haslund‐Vinding, Jaquet, & McBean, 2017). Antioxidant deficiencies within the SNc are therefore less likely to reflect reductions in neuronal antioxidant production and instead may signpost alterations to glial redox metabolism and/or regulation in aging and PD. Indeed, moderate age‐related microglial activation (Kanaan, Kordower, & Collier, 2010) and mild increases in reactive astrocytes (Jyothi et al., 2015) are documented in the postmortem primate and human SNc, and substantial microglial activation (Le, Wu, & Tang, 2016) and reactive astrogliosis (Booth, Hirst, & Wade‐Martins, 2017) are well‐documented hallmarks of the PD SNc. For both microglia and astrocytes, these changes are associated with PD‐linked gene mutations in familial PD or atypical alteration of their protein products in sporadic PD, which are demonstrated to impair glia‐derived antioxidant protection of SNc dopamine neurons (Joe et al., 2018). Astrocytes supply neurons with glutathione through a process regulated by Parkin, which becomes disrupted by *PARK2* mutations in familial PD. Astrocytes are also enriched in DJ‐1 protein, which scavenges ROS and induces antioxidant genes to protect neurons from oxidative insult (Bandopadhyay et al., 2004; Clements, McNally, Conti, Mak, & Ting, 2006). DJ‐1 modification or mutation in PD ameliorates this function (Bandopadhyay et al., 2004). Reduced antioxidant production by microglia may be attributed to a predominant M1 activation phenotype, associated with ROS production and pro‐inflammatory responses, rather than an antioxidant, anti‐inflammatory M2 phenotype (Rojo et al., 2014). Elevated levels of ROS paradoxically drive M1 microglial activation, suggesting a self‐perpetuating cycle of pro‐oxidant microglial activation may be established within the aging SNc. Importantly, age‐related microglial activation occurs preferentially in the vulnerable vSNc (Kanaan et al., 2010) and may therefore contribute to the selective vulnerability of this nigral subregion to oxidative insult in PD.

Improving our knowledge of the specific contributions of non‐neuronal cell types to physiological redox homeostasis in the SNc will undoubtedly advance our understanding of the origins of redox dyshomeostasis in healthy aging and PD pathogenesis. At present, our ability to make significant progress in this area is impaired by the limited availability of analytical technologies capable of resolving individual cells within animal or human tissues. The recent development of digital spatial profiling technology (NanoString) for cancer and immunology research (Gullo et al., 2018; Sun et al., 2011) opens remarkable possibilities for quantitative assessment and comparison of mRNA, protein, and small molecule expression profiles between cell types in neuroscience research.

## 7 ROS ACCUMULATION AND CELL DEATH PATHWAYS

It is clear that ROS accumulation is a primary feature of numerous damaging molecular pathways present during early‐stage PD, prior to initiation of neuron death. Excessive ROS accumulation can promote, or directly trigger, numerous cell death pathways including apoptosis (intrinsic and extrinsic), cytoplasmic cell death (parthanatos and necroptosis), and autophagic cell death (Morris, Walker, Berk, Maes, & Puri, 2018; Redza‐Dutordoir & Averill‐Bates, 2016), although technological limitations prevent us from determining which are activated in the SNc of PD patients in vivo. Further, the relatively slow rate of SNc dopamine neuron loss and rapid clearance of dead dopamine neurons by phagocytosis makes postmortem detection of cell death markers difficult and often unreliable. TUNEL staining, which labels DNA fragmentation as an indication of apoptosis, generates variable results in the PD SNc ranging from no TUNEL‐positive neurons to a high percentage of TUNEL‐positive neurons in PD and age‐matched controls (Levy, Malagelada, & Greene, 2009). More indirect approaches to identify and quantify the expression of cell death pathways in postmortem PD tissues have evaluated the expression of pathway constituents, including Bcl‐2 family proteins, caspases, Fas, and p55 (Jellinger, 2000; Mogi et al., 2000), with similar variable results and reproducibility. Compounding these issues, evidence of other cell death signaling pathways in PD patient tissues, such as markers of autophagic cell death, is difficult to interpret as they can signal processes responsible for either suppressing or promoting cell death (Levy et al., 2009). For these reasons, most research into which cell death pathways are activated in the PD SNc, and what their primary triggers are, has utilized dopaminergic cell cultures and animal models of PD. In these models, neuronal cell death associated with mutations in PD‐linked genes (*SNCA, LRRK2*, *DJ‐1, PARK2*, *PINK‐1)* involves varying degrees of activation of intrinsic (Iaccarino et al., 2007; Yamada, Iwatsubo, Mizuno, & Mochizuki, 2004) and extrinsic (Ho, Rideout, Ribe, Troy, & Dauer, 2009) apoptosis, autophagic cell death (Venderova & Park, 2012), and parthanatos (Kam et al., 2018). Similarly, intrinsic (Clayton, Clark, & Sharpe, 2005; Perier et al., 2005) and extrinsic (Hayley et al., 2004) apoptosis, necroptosis (Callizot, Combes, Henriques, & Poindron, 2019), and parthanatos (David, Andrabi, Dawson, & Dawson, 2009) are implicated in dopamine neuron death resulting from the administration of PD‐mimetic toxins MPTP, rotenone, and 6‐OHDA, albeit to differing extents.

Discerning the role of ROS in activating and/or accelerating these pathways adds another layer of complexity, requiring a clear relationship between a ROS levels and pathway activation to be established. Given the relatively new discovery of pathways such as parthanatos, or the recent evolution of pathways such as necroptosis from the coalescence of existing cell death mechanisms, there has been little investigation of a specific role for ROS in dopamine neuron death mediated by these pathways. Increased ROS production accompanied neuron death in MPTP‐, rotenone‐, and 6‐OHDA‐treated primary cultures of rat mesencephalic dopamine neurons, where there was also evidence of significant parthanatos (MPTP, rotenone, 6‐OHDA) and necroptosis (MPTP, rotenone; Callizot et al., 2019). While ROS accumulation is indeed known to trigger both of these pathways (Morris et al., 2018), it is unknown whether antioxidant‐mediated attenuation of ROS levels reduces the activation of these pathways following toxin administration; the final step required to confirm causality. It would also be a valuable extension to see such an experiment repeated in a more complex mammalian model system, where the influences of other non‐neuronal cell types implicated in the toxicity of PD‐linked agents would be present. Contrast to parthanatos and necroptosis, a strong body of data implicates ROS accumulation in the priming, initiation and acceleration of apoptosis in SNc dopamine neurons in cell and animal models of PD. An abundance of evidence implicates mitochondrially driven (intrinsic) apoptosis, in particular, to ROS‐driven cell death in PD (Venderova & Park, 2012), likely linked to well‐documented mitochondrial dysfunction reported in degenerating brain regions in PD.

### 7.1 Mitochondria‐dependent (intrinsic) apoptosis

The intrinsic (mitochondrial) cell death pathway is activated by cellular stressors, including excessive ROS accumulation, which trigger conformational change and mitochondrial localization of pro‐apoptotic Bcl‐2 family proteins (Bax, Bak, Bad, Bim). These proteins oligomerize in the outer mitochondrial membrane and form pores that alter mitochondrial membrane permeability, facilitating the release of pro‐apoptotic proteins from the mitochondrial intermembrane space, including cytochrome c, second mitochondria‐derived activator of caspase (Smac) endonuclease G, and apoptosis‐inducing factor (AIF; Figure 5; Perier & Vila, 2012). Cytosolic cytochrome c interacts with Apaf‐1 and procaspase‐9, which together activate executioner caspases (caspase‐3) that trigger apoptosis by proteolytic cleavage of numerous key cellular proteins. Smac likewise activates caspases by inhibiting cytoplasmic caspase inhibitor proteins (IAPs), whereas AIF released from mitochondria translocates to the nucleus to trigger chromatin condensation and DNA fragmentation, inducing cell death. Anti‐apoptotic Bcl‐2 family proteins (Bcl‐2, Bcl‐xL), on the other hand, bind to activated pro‐apoptotic Bcl‐2 proteins and hinder their oligomerization, preventing the release of pro‐apoptotic factors from mitochondria. Excessive ROS accumulation causes transcriptional downregulation of anti‐apoptotic Bcl‐2 proteins and upregulation of pro‐apoptotic Bcl‐2 proteins, mediated by ROS‐induced conformational change in cytosolic p53 (Redza‐Dutordoir & Averill‐Bates, 2016). This also triggers the mitochondrial localization of p53, where it directly binds to and activates Bax and other pro‐apoptotic Bcl‐2 proteins and blocks anti‐apoptotic Bcl‐2 proteins (Luna‐Vargas & Chipuk, 2016). ROS‐induced activation of cytosolic c‐Jun N‐terminal kinase (JNK) similarly results in Bcl‐2 inhibition and Bim/Bad activation (West & Marnett, 2006). Together these proteins constitute key effectors regulating intrinsic apoptosis.

Evidence of intrinsic apoptosis is present in numerous chemical and genetic models of PD with well‐characterized ROS accumulation. A time‐dependent, region‐specific release of cytochrome c from mitochondria is observed in low‐mid dose rotenone‐ (Clayton et al., 2005) and MPTP‐treated (Perier et al., 2005) mice, followed by activation of caspase‐9, caspase‐3, and apoptotic nigral cell death (Perier et al., 2005). Overexpression of mutant α‐synuclein in vivo and in mice triggers caspase‐dependent apoptosis of dopaminergic neurons (Yamada et al., 2004), associated with p53 activation (Martin et al., 2006) and cytochrome c release from mitochondria (Parihar, Parihar, Fujita, Hashimoto, & Ghafourifar, 2008). Mutations in *LRRK2*, *PARK2,* and *PINK‐1* are associated with mitochondria‐dependent apoptosis in vitro and in murine models of PD*,* through p53 activation (Ho, Seol, & Son, 2019), cytochrome c release, and caspase activation (Iaccarino et al., 2007). Importantly, there is clear evidence of a primary role for ROS in driving the intrinsic apoptosis pathway in these models; overexpression of SOD1 or glutathione peroxidase, or administration of exogenous glutathione, reduced ROS accumulation and prevented toxin‐ and α‐synuclein‐induced intrinsic apoptosis in SNc dopamine neurons (Flower, Chesnokova, Froelich, Dixon, & Witt, 2005; Przedborski et al., 1992; Smeyne & Smeyne, 2013; Thiruchelvam et al., 2005). Further, selegiline‐induced neuroprotection of 6‐OHDA‐treated neurons derives from the upregulation of SOD1 and catalase expression, and the suppression of pro‐oxidant, iron‐catalyzed dopamine auto‐oxidation (Ghavami et al., 2014; Khaldy et al., 2000). More specifically, excessive ROS appears to play a pivotal role in both priming and triggering apoptosis in these models, depending on the subcellular localization of ROS accumulation (Figure 5). Aside from activating cytosolic p53/JNK/Bax/Bak to directly trigger intrinsic apoptosis, ROS generated by MPTP and rotenone damage cardiolipin within the mitochondrial ETC, which normally anchors cytochrome c in the ETC between complexes III and IV (Rytomaa & Kinnunen, 1995). Cytochrome c is subsequently released into the mitochondrial intermembrane space (Petrosillo, Ruggiero, Pistolese, & Paradies, 2001), elevating the releasable soluble pool of cytochrome c and priming mitochondria for substantial cytochrome c release following p53/JNK/Bax/Bak activation by cytosolic ROS or other cellular stressors (Perier et al., 2005). The accumulation of ROS both within and outside of mitochondria therefore constitutes an important sensitizer and inducer of the intrinsic apoptosis pathway (Redza‐Dutordoir & Averill‐Bates, 2016). While most work in this area has been conducted in toxin‐based PD models, which possess comparatively low physiological relevance, the accumulation of pathogenic PD‐linked proteins (α‐synuclein), deficiency of protective PD‐linked proteins (DJ‐1), or the incorporation of PD‐linked mutations (*SNCA*) similarly result in ROS‐dependent apoptosis in cultured human or rat dopaminergic neurons (Kim et al., 2005; Xu et al., 2002). Common mechanisms of ROS‐dependent apoptosis may therefore connect multiple established pathogenic pathways in the PD SNc, irrelevant of their chemical or genetic bases. Future research should be directed toward characterizing the importance of ROS in apoptosis and other cell death pathways (necroptosis, parthanatos) implicated in SNc dopamine neuron death in PD, and to developing technologies capable of measuring their biomarkers in animal models of PD and patient tissues.

A lowered threshold for the intrinsic pathway of apoptosis may help to explain why severe nigral dopamine neuron loss occurs in PD but is avoided in the healthy aged brain. Age‐related pathological change imparts little functional consequence during healthy aging itself, where a higher threshold exists for activation of the intrinsic apoptotic pathway and redox imbalance in the SNc is comparatively mild. By contrast, environmental and/or genetic factors associated with PD pathogenesis lower the activation threshold for apoptosis and generate substantial ROS specifically within the SNc, triggering apoptosis of nigral dopamine neurons.

### 7.2 Apoptosis as a therapeutic target

Translating insights from mechanisms of cell death into therapies capable of slowing or halting cell death in PD poses several challenges. Ironically, cell death pathways can form part of normal physiological responses. For example, cell death mechanisms are utilized to prevent processes such as tumor growth. Other data suggest that, independent of the cell death pathway targeted, cells at risk may eventually die by alternative mechanisms (Hartmann et al., 2001) driven by primary pathogenic factors upstream, including oxidative stress. Multiple cell death pathways may also be activated within a given neuron (Callizot et al., 2019), and degeneration within different neuronal compartments (axon, soma) may be governed by different pathways (Ries et al., 2008). Further, blockade of penultimate cell death pathway components (caspases, Apaf‐1) may prevent neuron death but not preserve or improve cellular functions disrupted by primary pathogenic factors (Levy et al., 2009). Such considerations argue disease‐modifying therapies should target primary upstream mechanisms, such as ROS accumulation, to prevent the activation and/or acceleration of cell death pathways.

## 8 TARGETING OXIDATIVE STRESS AS A THERAPEUTIC MODALITY

Despite a clear contribution of ROS accumulation to the initiation and/or acceleration of cell death in PD, numerous large and well‐conducted clinical trials targeting oxidative stress in this disorder have shown little improvement in clinical outcome for patients (Table 1). Approximately eleven double‐blind, placebo‐controlled randomized clinical trials of antioxidants have been completed in PD patients who were within 5 years of diagnosis or less, targeting multiple biochemical pathways for durations ranging between 1 month and 8 years. No evidence of clinical benefits, as measured by improvements in unified Parkinson's disease rating scale (UPDRS) scores, were observed despite these wide and varied approaches. However, rather than immediately adopt the pessimistic perspective that therapies targeting oxidative stress in PD are inherently flawed in concept, we should first consider some of the reasons this may be so (Murphy, 2014).

**Table 1:** Summary of antioxidant clinical trials for Parkinson's disease

Authors	Study design	Participants	Intervention/duration	Redox modulation	Outcome measures	Status/results
The Parkinson Study Group and DATATOP study (1993)	DPRCT	800 early PD patients, within 5 years of diagnosis	2‐year intervention Randomization Deprenyl (10 mg/day) α‐tocopherol (2,000 IU/day) Deprenyl/α‐tocopherol Placebo	Deprenyl—reduced toxin‐induced ^●^OH^−^ formation (Wu, Chiueh, Pert, & Murphy, 1993), and iron‐induced oxidative stress (Budni et al., 2007). α‐Tocopherol—lipophilic ROS scavenger, reduced lipid peroxidation (Niki, 2015)	The onset of disability prompting levodopa administration	Complete—no evidence of clinical benefit
Shults et al. (2002)	DPRCT	80 early PD patients who did not require treatment for their disability	16‐month intervention Randomization Coenzyme Q (300 mg/day) Coenzyme Q (600 mg/day) Coenzyme Q (1,200 mg/day) Placebo	Coenzyme Q—lipophilic ROS scavenger, reduces lipid peroxidation (Bentinger, Brismar, & Dallner, 2007)	Safety and tolerability, UPDRS score	Complete –safe and tolerable up to 1,200 mg/day, appeared to slow progression of PD motor impairment
The Parkinson Study Group	DPRCT	600 early PD patients, within 5 years of diagnosis	16‐month intervention Randomization Coenzyme Q (1,200 mg/day) Coenzyme Q (2,400 mg/day) Placebo Each group combined with α‐tocopherol (1,200 IU/day)	Coenzyme Q and α‐Tocopherol as above	UPDRS score	Complete—no evidence of clinical benefit
Snow et al. (2010), Protect Study Group	DPRCT	128 early PD patients who do not require treatment for their disability	12‐month intervention Randomization MitoQ (40 mg/day) MitoQ (80 mg/day) Placebo	MitoQ—a coenzyme Q mimetic, mitochondria‐targeted ROS scavenging (Tauskela, 2007)	UPDRS score	Complete—no evidence of clinical benefit
NET‐PD investigators	DPRCT	1,741 early PD patients, within 5 years of diagnosis	5‐ to 8‐year intervention Randomization Creatine (10g/day) Placebo	Creatine—induction of antioxidant enzymes (TRx, PRx), effective scavenger of ^●^OH^−^ and O_2_^−^, protects against mitochondrial DNA and RNA (Sestili et al., 2011)	Modified Rankin Scale, Symbol Digit Modalities Test, PDQ−39 Summary Index, Schwab and England Activities of Daily Living scale, and ambulatory capacity	Terminated early for futility—no evidence of clinical benefit
Mischley, Lau, Shankland, Wilbur, and Padowski (2017)	DPRCT	45 early PD patients (H&Y stage 1–3)	3‐month intervention Randomization GSH (300 mg IN/day) GSH (600 mg IN/day) Placebo	GSH—low molecular weight antioxidant; ROS detoxification, redox signaling molecule, substrate for antioxidant enzyme pathways (Forman, Zhang, & Rinna, 2009)	UPDRS	Completed—no evidence of clinical benefit
Hauser, Lyons, McClain, Carter, and Perlmutter (2009)	DPRCT	21 PD patients (H&Y stage 2–5), nonresponsive to L‐DOPA	4‐week intervention Randomization GSH (1,400 mg IV, 3×/week) Placebo	GSH as above	Safety and tolerability, UPDRS	Completed—safe and tolerable up to 4,200 mg/week, no preliminary evidence of clinical benefit
NINDS Exploratory Trials in Parkinson Disease	DPRCT	210 early PD patients, within 5 year of diagnosis (H&Y stage 1–2)	44‐week intervention Randomization Pioglitazone (15 mg/day) Pioglitazone (45 mg/day) Placebo	Pioglitazone—induction of peroxisomal and cytosol antioxidant proteins (SOD1, catalase, GPx1; Filograna et al., 2016)	UPDRS	Completed—no evidence of clinical benefit
Weill Medical College of Cornell University (NCT01470027)	Parallel assignment DPRCT	30 PD patients, less than 15 years postdiagnosis	4‐week intervention Randomization N‐acetyl‐cysteine (1,800 mg/day) N‐acetyl‐cysteine (3,600 mg/day) Placebo	*N*‐acetyl‐cysteine—ROS scavenger and major substrate for GSH/GPx antioxidant pathway (Firuzi et al., 2011)	UPDRS, Cerebral GSH levels (measured by Proton Magnetic Resonance Spectroscopy)	Completed—results yet to be published
NINDs, Michael J. Fox Foundation, The Parkinson Study Group (NCT02168842)	Parallel assignment DPRCT	336 early PD patients, within 3 years of diagnosis (H&Y stage 1–2)	3‐year intervention Randomization Isradipine (10 mg/day) Placebo	Isradipine—Calcium channel antagonist, reduction in mitochondrial ROS production by reducing calcium‐buffering burden (Rodnitzky, 1999)	UPDRS	Active—results yet to be published
Martin‐Bastida et al. (2017)	DPRCT	22 early PD patients, within 5 years of diagnosis (H&Y stage 1–2)	6‐month intervention Randomization Deferiprone (20 mg kg day^−1^) Deferiprone (30 mg kg day^−1^) Placebo	Deferiprone—effective iron chelator, reduces iron‐mediated ROS generation (Hare & Double, 2016)	Safety and tolerability, UPDRS, brain iron concentrations assessed by T2* MRI	Completed—safe and tolerable up to 30 mg kg day^−1^, no evidence of clinical benefit
FAIR PARK II (NCT02655315)	Parallel assignment DPRCT	338 early PD patients, within 18 months of diagnosis, (H&Y stage 1–3)	9‐month intervention Randomization Deferiprone (30 mg kg day^−1^) Placebo	Deferiprone as above	UPDRS	Recruiting
Collaborative Medicinal Development Pty Limited (NCT03204929)	Single group assignment, open label dose escalation	38 PD patients (estimated), within 5 years of diagnosis (H&Y stage 1–2)	6‐month intervention of Cu(II)‐atsm Dose escalation (starting 12 mg/day) to determine RP2D. Phase 2—expansion cohort (*n* = 20 patients) treated with RP2D until 6 months	Cu(II)‐atsm—reduction of cellular ROS and peroxynitrite, largely unknown mechanism, promotion of SOD1 antioxidant activity (Hung et al., 2012)	Safety and tolerability – patients in each dose cohort with intolerance up to 6 months treatment, UPDRS	Recruiting

Abbreviations: DPRCT, double‐blind, placebo‐controlled, randomized clinical trial; H&Y, Hoehn and Yahr; PD, Parkinson's disease; UPDRS, Unified Parkinson's Disease Rating Scale.

As a primary outcome, clinical trials must assess whether potential therapies slow or halt PD progression in patients by eliciting a significant reduction in SNc dopamine neuron loss, a significant challenge given the absence of reliable techniques for quantifying dopamine neurons in vivo. *In lieu* of such technology, we are forced to employ indirect measures of dopamine neuron health and number, most commonly involving an assessment of motor function using scales such as the UPDRS (Table 1). While the UPDRS constitutes a widely employed metric of PD severity, it is not without limitation; some items within the UPDRS exhibit poor inter‐rater reliability, others may be influenced by common age‐related comorbidities, and there is concern that some items no longer reflect conceptual thinking about PD (Movement Disorder Society Task Force on Rating Scales for Parkinson's Disease, 2003). Most importantly, the UPDRS assumes any measured movement dysfunction derives from dopaminergic denervation, and is thus a relatively blunt instrument in its resolution of SNc‐specific neurodegeneration. Alternatives to the UPDRS, including in vivo imaging of dopamine receptors or transporters using radioligands (Loane & Politis, 2011; Niccolini, Su, & Politis, 2014; Shih, Hoexter, Andrade, & Bressan, 2006), are also widely employed in clinical trials to assess nigral dopaminergic neurotransmission, although it is unclear whether these measurements accurately correlate with dopaminergic neuron density in human PD patients. Until we develop reliable methods for quantifying SNc dopamine neurons in vivo, we cannot be sure whether the apparent failures of many well‐conducted trials of antioxidants or other therapies in PD patients derives from a lack of treatment efficacy or our inability to detect alterations in PD progression.

Aside from our inability to quantify dopamine neurons in vivo, the timing of therapeutic administration is likely to have a substantial influence on the efficacy of antioxidants in clinical trials for PD. Data from PD patients and models indicate redox dyshomeostasis within the SNc is both an initiating and driving factor in PD pathogenesis, suggesting restoration of redox buffering capacity in SNc dopamine neurons would yield the greatest therapeutic effect when administered prophylactically (Firuzi, Miri, Tavakkoli, & Saso, 2011). Most clinical trials have assessed the efficacy of antioxidants in patients who received a clinical diagnosis of PD within the last 5 years (Table 1); however, even at the time of diagnosis approximately 50%–70% of SNc dopamine neurons have been lost (Cheng, Ulane, & Burke, 2010). It is plausible that the efficacy of many promising antioxidant therapies may well be below detectable levels in these patients, due to the onset of significant clinical symptoms and the initiation of later phases of PD neurodegeneration, which may now progress independently of initiating primary pathogenic factors. The recruitment PD patients who are within this timeframe of diagnosis is therefore regrettable but is completely understandable given the absence of any reliable biomarkers for identifying PD patients in preclinical stages of the disorder, when motor symptoms have not yet manifested. Significant attention is therefore being focused toward developing a means of identifying PD in its preclinical stages using blood or CSF‐based molecular biomarkers, or to similarly identify individuals who are at a high risk of conversion to PD in the clinic. Idiopathic rapid eye movement sleep behavior disorder (iRBD), for example, is among the most common early signs of PD and is increasingly being recognized as a powerful opportunity to observe PD in its prodromal stages. Recent data from a large multicentre cohort of iRBD patients (*n* = 1,280) estimate a 15.8% phenoconversion rate to PD within 4.6 years of baseline examination (Postuma et al., 2019) and suggest a mixture of motor (UPDRS) and cognitive (office‐based cognitive testing, neuropsychological examination, color vision testing) variables may enable stratification of prodromal PD patients from other iRBD individuals for recruitment into neuroprotective trials for PD. The reliable identification of individuals with prodromal PD will enable the administration of promising therapies targeting primary etiological factors at a point within the disease process likely to impart the greatest neuroprotection.

An abundance of evidence from genetic and toxin‐induced animal models of PD demonstrates redox imbalance contributes to SNc neurodegeneration, and further, that restoring redox homeostasis with these neurons protects against degeneration (Biosa et al., 2018; Devos et al., 2014; Dias et al., 2013; Filograna, Beltramini, Bubacco, & Bisaglia, 2016; Zhou & Freed, 2005). These data, however, are made possible by our ability to carefully dissect and assay brain tissue from these animals to profile cellular ROS and antioxidant function, a workflow that is clearly not translatable to patients in clinical trials. Moreover, similar to our inability to quantify dopamine neurons in vivo, current technological limitations prevent us from assaying nigral ROS, oxidative damage, or even drug penetrance *in* vivo; thus, most trials to date have relied upon the UPDRS (Table 1), a clinical scale for disease severity and a rather blunt instrument not designed for empirical research. Results from clinical trials are therefore often difficult to interpret; is a lack of success derived from improper delivery, inadequate dosage or duration, or total lack of antioxidant effect? The development of reliable in vivo imaging techniques capable of quantifying levels of cellular ROS and/or the function of cellular antioxidants will be key to future assessments of the therapeutic efficacy of antioxidant therapies in clinical trials for PD.

Many antioxidant therapies trialed to date also have inherent limitations that may restrict their usefulness in central nervous system disorders. Several promising antioxidant compounds, including coenzyme Q, creatine, and deferiprone, have poor brain penetration (Abbruzzese et al., 2011; Hanna‐El‐Daher & Braissant, 2016; Rotig, Mollet, Rio, & Munnich, 2007) and therefore may not enter the brain in sufficient quantities at tested doses to efficiently detoxify ROS. Upon entering the brain, many compounds disperse relatively homogenously throughout tissues and cells (Murphy, 2014), a distribution in stark contrast to the cell‐ or organelle‐specific localization of ROS accumulation. As a consequence, the total antioxidant capacity of a given tissue or cell may be sufficient to protect from global ROS‐induced damage, but localized antioxidant concentrations within cellular, or subcellular, ROS hotspots may be insufficient to prevent ROS accumulation and oxidative damage. Recognition and acknowledgment of these limitations may help to guide the development of improved antioxidant therapies with enhanced bioavailability and compartmental specificity. Just as pharmacological interventions targeting the aggregation of specific proteins are often highly specific to their target, antioxidant therapies might be most beneficial when designed to selectively interact with an oxidative target or process within a specific subcellular compartment (Murphy, 2014). Although an early clinical trial of the mitochondria‐targeted antioxidant, MitoQ, in early‐stage PD did not report clinical benefits (Table 1), Szeto‐Schiller (SS) peptides represent another approach to target antioxidant activity directly to mitochondria. Szeto‐Schiller peptides readily cross the blood–brain barrier and have demonstrated in vivo antioxidant efficacy in murine models of PD (Szeto & Schiller, 2011). While their potential to slow or halt progression of PD has not been investigated to date, a phase 2 double‐blind, placebo‐controlled, randomized clinical trial of SS‐31 (Elamipretide) is currently underway in subjects with age‐related macular degeneration (ClinicalTrials.gov; NCT03891875), following promising data regarding safety and tolerability in individuals with primary mitochondrial myopathy (Karaa et al. (2018), ClinicalTrials.gov; NCT02367014).

A final growing criticism of most therapies trialed in PD patients is their relative simplicity compared with the complex degenerative cascade underlying neuron death in PD. Endogenous antioxidants function within an integrated and coordinated network, combining the actions of numerous small molecules with the enzymatic activities of many proteins. Monotherapeutic antioxidant treatment regimes trialed in the clinic rarely address more than a single target. Combined administration of multiple antioxidant therapies influencing multiple network targets or pathways may therefore impart a greater therapeutic benefit and should be considered for future trials of antioxidants. Further, while ROS accumulation plays a key role in the initiation and acceleration of cell death in PD, it is not the sole origin of cell death in this disorder. A multifaceted treatment approach simultaneously targeting ROS accumulation and additional PD‐linked pathologies, such as α‐synuclein deposition or calcium dysregulation, may possess a greater potential to slow or halt disease progression.

## CONFLICT OF INTEREST

None declared.
